# Prosthetic Rehabilitation With a Tooth-Supported Overdenture Utilizing Ball Attachments and a Customized Intraoral Tracer: A Case Report

**DOI:** 10.7759/cureus.96177

**Published:** 2025-11-05

**Authors:** Velpula Hima Varshini, Savitha PN, Krishnaprasad TR, Vedashri R Sakhare, Ananthesh HS

**Affiliations:** 1 Prosthodontics and Crown and Bridge, The Oxford Dental College, Bangalore, IND; 2 Prosthodontics, The Oxford Dental College, Bangalore, IND

**Keywords:** balanced occlusion, ball attachment, case report, intra oral tracer, overdentures

## Abstract

The preservation of the residual ridge remains a fundamental objective in prosthodontic rehabilitation, particularly following tooth extraction. Retaining two or more natural teeth as abutments facilitates effective management of partially edentulous arches. Tooth-supported overdentures, as part of preventive prosthodontics, aid in conserving natural teeth, preserving proprioception, and minimizing alveolar bone resorption. Additionally, establishing a balanced occlusion further enhances denture stability and reduces ridge resorption. A 60-year-old male patient presented with difficulty in mastication due to the loss of multiple teeth in both the maxillary and mandibular arches. Clinical evaluation showed total edentulism in the maxillary arch, whereas the mandibular arch exhibited partial edentulism with the presence of teeth 33, 43, and 44. The proposed treatment plan emphasized preservation of the remaining dentition and prosthetic restoration using a conventional complete denture for the maxillary arch and a mandibular overdenture supported by retained teeth with ball attachments. A customized intraoral tracer was utilized to achieve precise jaw relation records, and balanced occlusion was incorporated to optimize prosthetic function and ridge preservation.

This report highlights that tooth-supported overdentures offer significant advantages in preserving the residual ridge and enhancing the functional and psychological outcomes of prosthodontic rehabilitation. The combined use of a customized intraoral tracer and balanced occlusion significantly enhanced the overall retention, support, and function of the prosthesis.

## Introduction

Residual ridge resorption is a continuous and progressive phenomenon following the loss of natural teeth. This gradual bone reduction compromises the stability and support of conventional dentures. As DeVan aptly stated, “The perpetual preservation of what remains is more important than the meticulous replacement of what is missing” [1]. The preservation of existing dentition, although often overshadowed, remains a crucial treatment objective. Every effort should be made to conserve natural tooth structure [2]. Retaining strategically positioned teeth under an overdenture helps preserve alveolar bone height and contour by maintaining proprioceptive feedback and functional load transmission, thereby enhancing long-term prosthesis stability. Overdentures, as a preventive prosthodontic approach, have proven effective in preserving alveolar bone and maintaining proprioceptive sensory feedback from retained tooth or roots.

Overdenture is defined as ‘any removable dental prosthesis that covers and rests on one or more remaining natural teeth, the roots of natural teeth, and/or dental implants’ [3]. Overdentures are of two types: tooth or implant-supported overdentures. Tooth-supported overdentures offer a conservative and economical treatment option, eliminating invasive surgical procedures. Clinical evidence suggests that a patient rehabilitated with a tooth-supported overdenture exhibits enhanced oral tactile sense due to preserved periodontal proprioception. Several overdenture designs exist, with tooth-supported types incorporating different attachment mechanisms being among the most widely utilized [4].

Ball attachments, which are stud-type connectors, are commonly used in overdenture designs to improve retention and stability. During the fabrication of a complete denture, accurate recording of maxillo-mandibular relation is essential for achieving optimal function, esthetics, and phonetics. Any error in this stage compromises the fit and comfort of the final dentures. Centric relation can be established using either graphic methods (extraoral or intraoral Gothic arch tracing) or physiological techniques. The use of a tracing device, such as the Gothic arch tracer (a central bearing device), provides a reliable means of recording centric relation. Among these methods, the intraoral tracer, a device that records centric relation by tracing mandibular movements, offers superior accuracy thanks to its proximity to the condylar rotational axis. To enhance prosthesis retention and stability, balanced occlusion, characterized by simultaneous bilateral tooth contact during mandibular excursions, helps distribute occlusal forces evenly and minimizes denture tipping. The integration of a customized intraoral tracer and balanced occlusion significantly enhanced the overall retention, support, and function of the prosthesis [5].

This case report aims to describe the prosthetic rehabilitation of a patient using a mandibular tooth-supported overdenture with stud-type ball attachments, emphasizing the use of a customized intraoral tracer for precise centric relation recording and the establishment of balanced occlusion. The report highlights a simplified and predictable clinical approach that can be applied in similar cases to enhance prosthesis function and patient comfort.

## Case presentation

**Figure 1:**
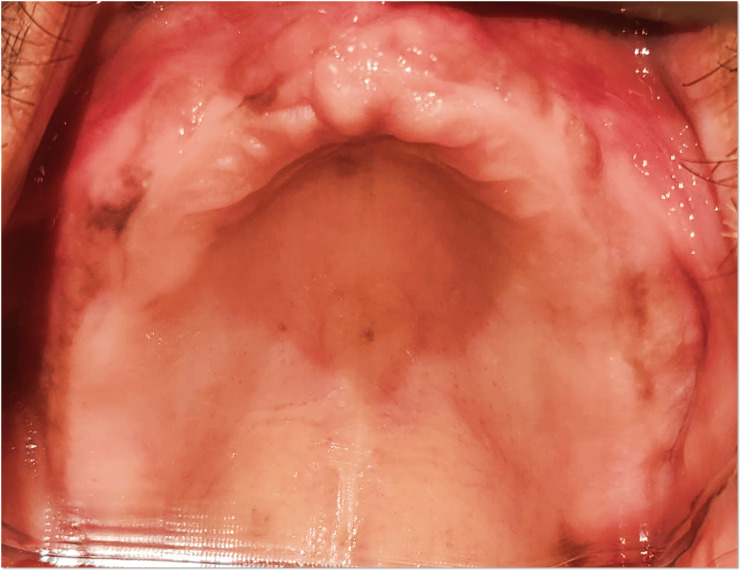
Maxillary arch - preoperative clinical view

**Figure 2:**
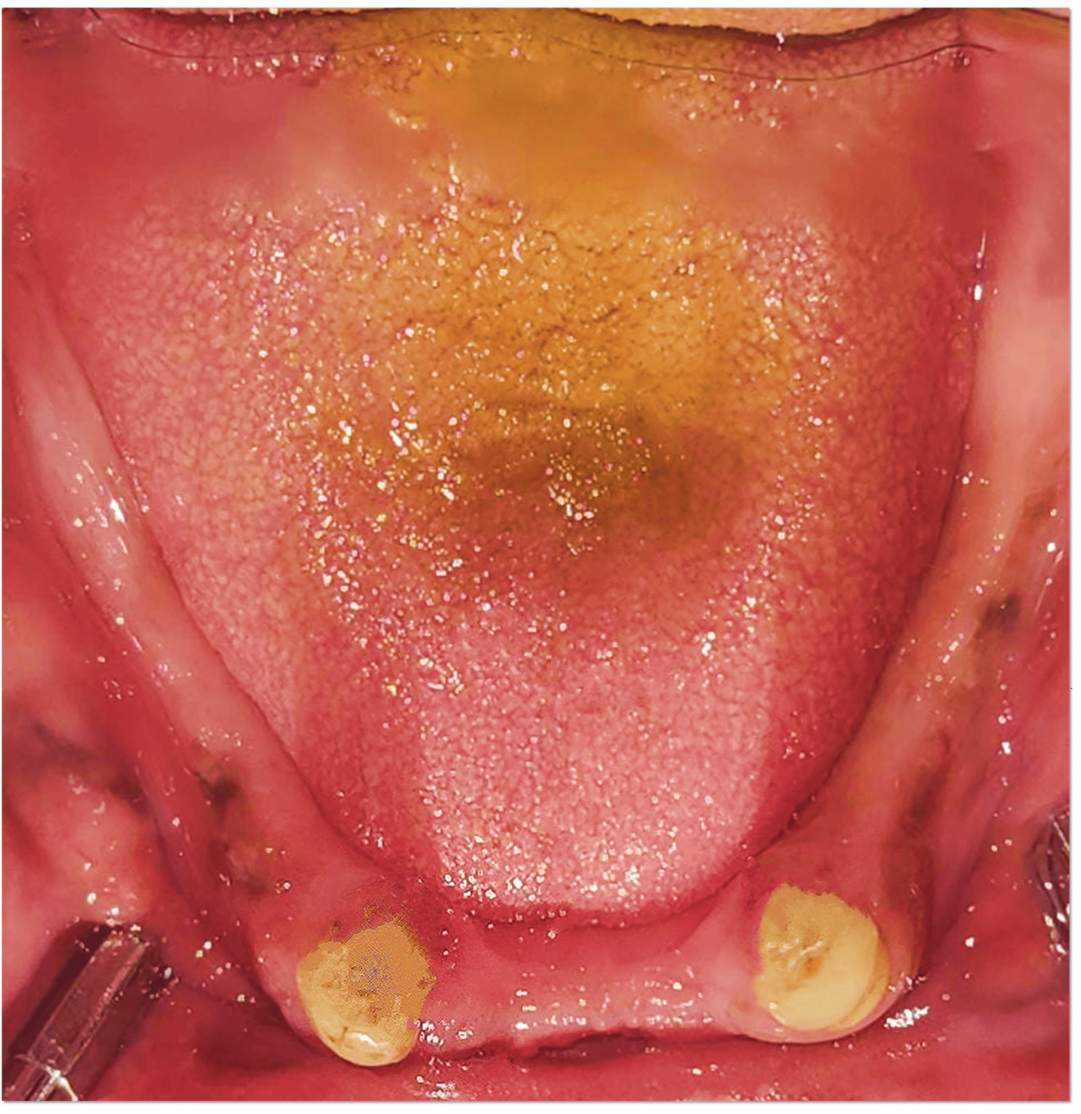
Mandibular arch - preoperative clinical view

**Figure 3:**
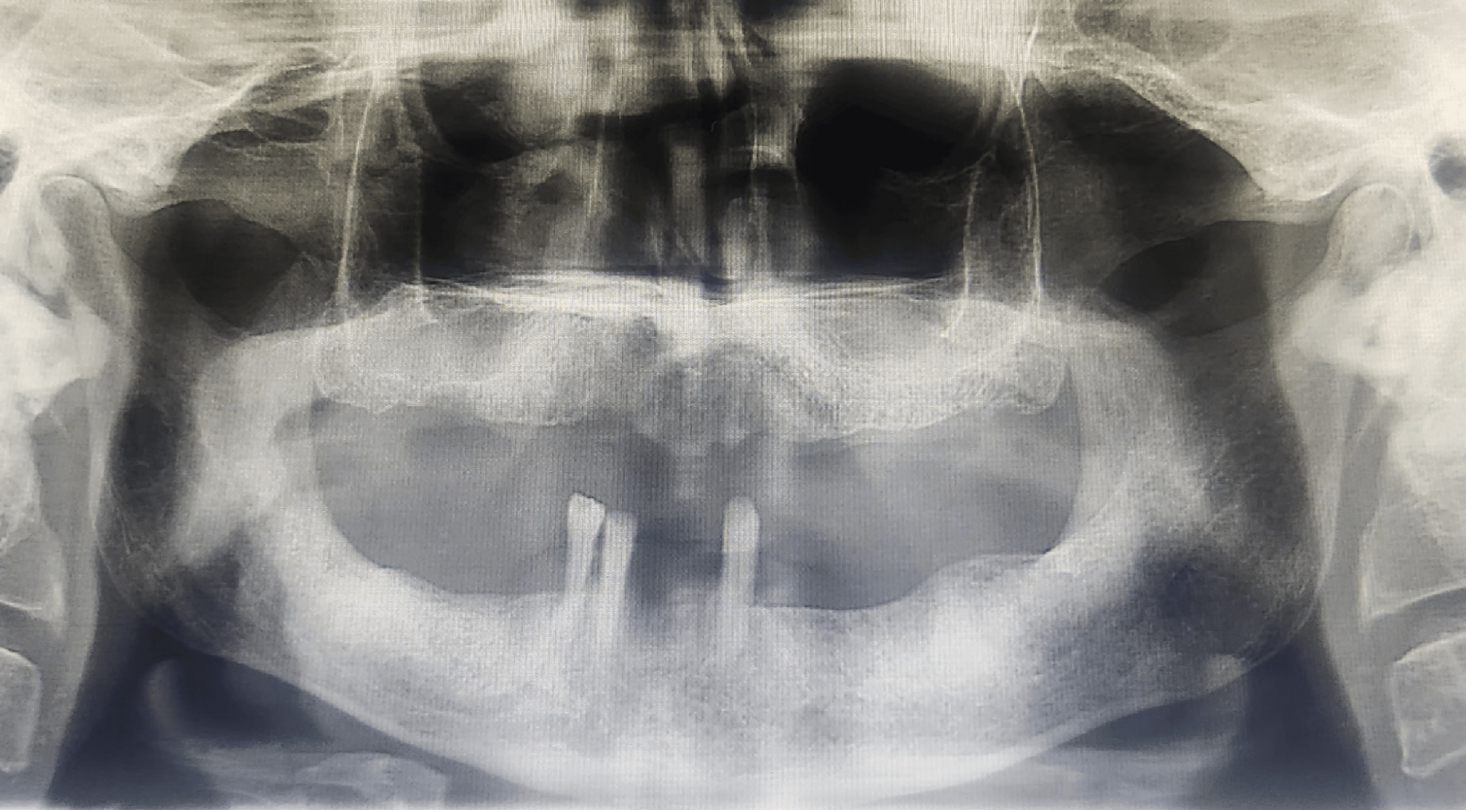
Orthopantomogram (OPG)

A 60-year-old male patient presented to the Department of Prosthodontics at The Oxford Dental College with the chief complaint of difficulty in mastication due to the loss of multiple teeth in both the maxillary and mandibular arches. The patient’s history revealed that the tooth loss occurred four years ago due to periodontal conditions. Clinical evaluation showed total edentulism in the maxillary arch (Figure 1), whereas the mandibular arch exhibited partial edentulism, with teeth 33, 43, and 44 remaining (Figure 2). Abutment assessment indicated that 33 and 43 were periodontally sound, while 44 exhibited Grade II mobility. Radiographic examination was conducted to assess the periodontal status and bone support of the remaining teeth (Figure 3).

Based on the clinical and radiographic findings, a treatment plan was developed. Given the favorable stability and periodontal condition of teeth 33 and 43, they were chosen to serve as abutments for a mandibular tooth-supported overdenture. Due to the compromised prognosis of tooth 44, extraction was advised. Ball attachments were planned for 33 and 43 to enhance the retention and stability of the overdenture. A conventional complete denture was planned for the maxillary arch to achieve optimal aesthetics, function, and patient comfort. The patient was informed of the treatment options, and consent was obtained before proceeding.

**Figure 4:**
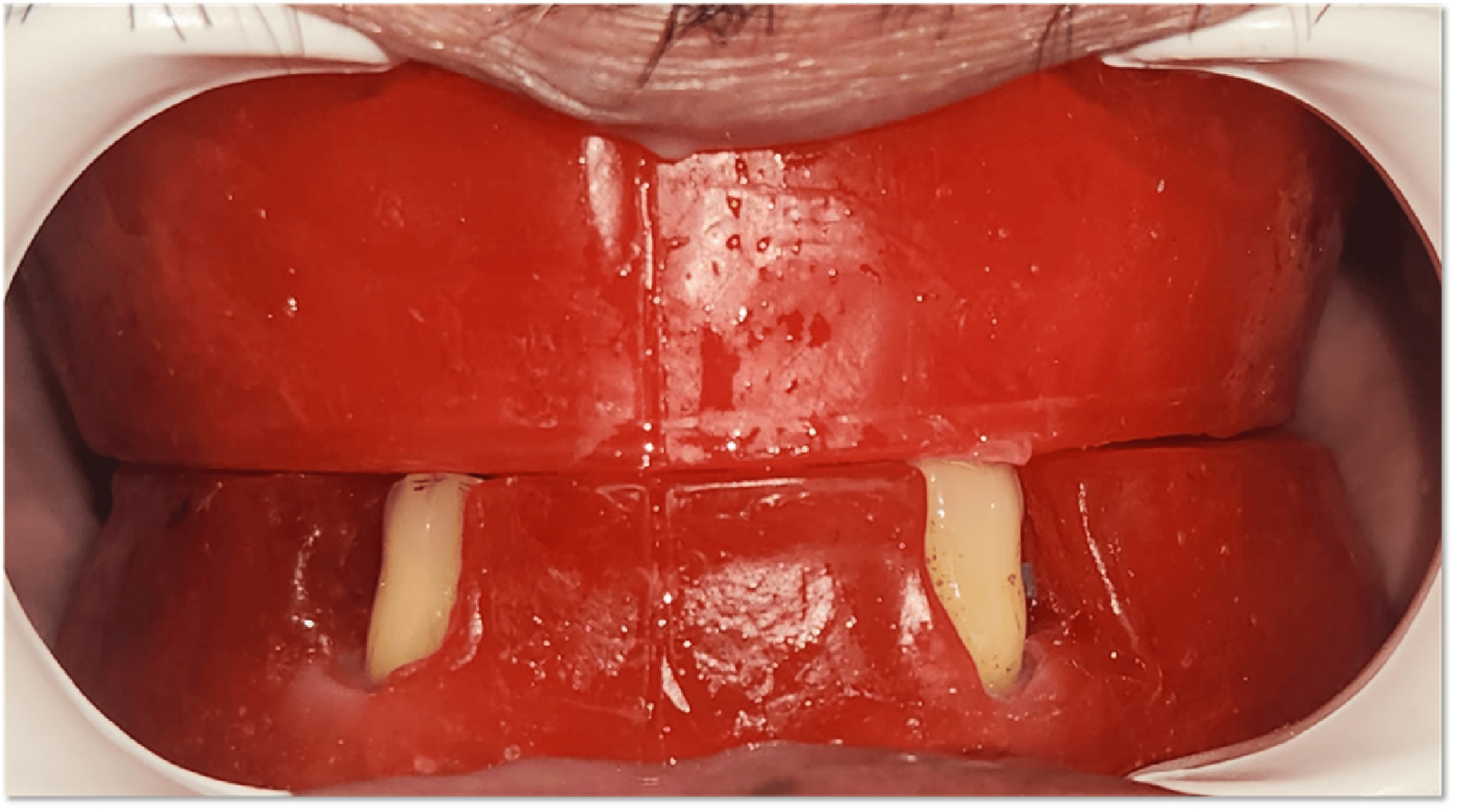
Tentative jaw relation record

After endodontic treatment, diagnostic impressions of both the maxillary and mandibular arches were made, and a tentative jaw relation record was established to check interocclusal space (Figure 4). Sufficient interarch space was available to accommodate ball attachments.

**Figure 5:**
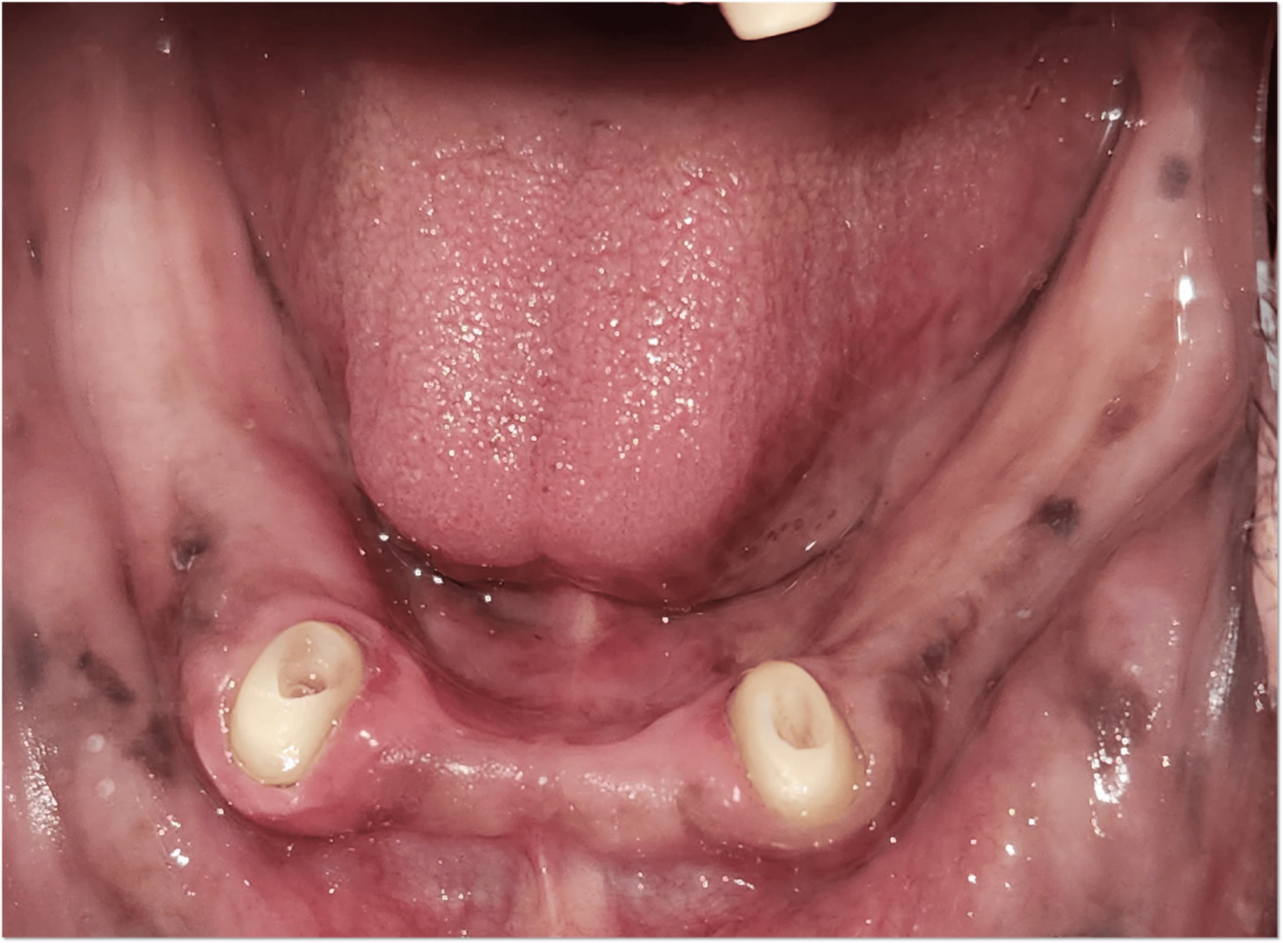
Post space preparation i.r.t., 33 and 43

**Figure 6:**
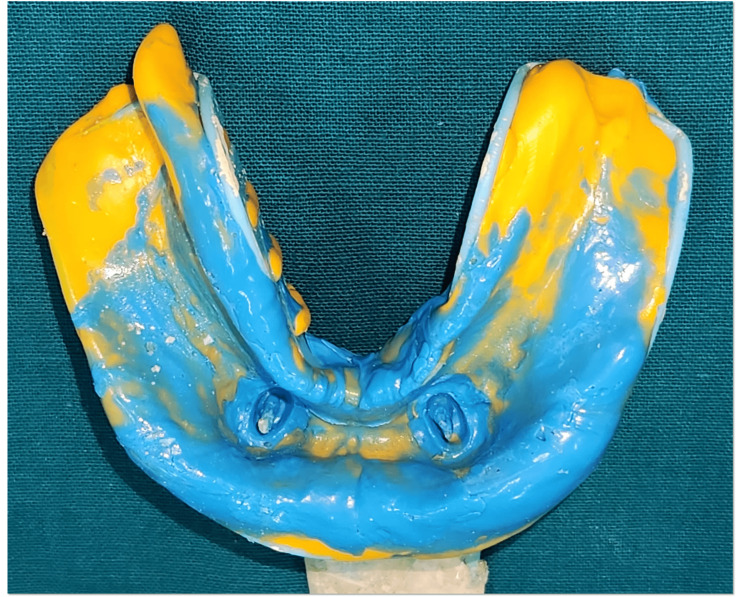
Final impression of the mandibular arch

**Figure 7:**
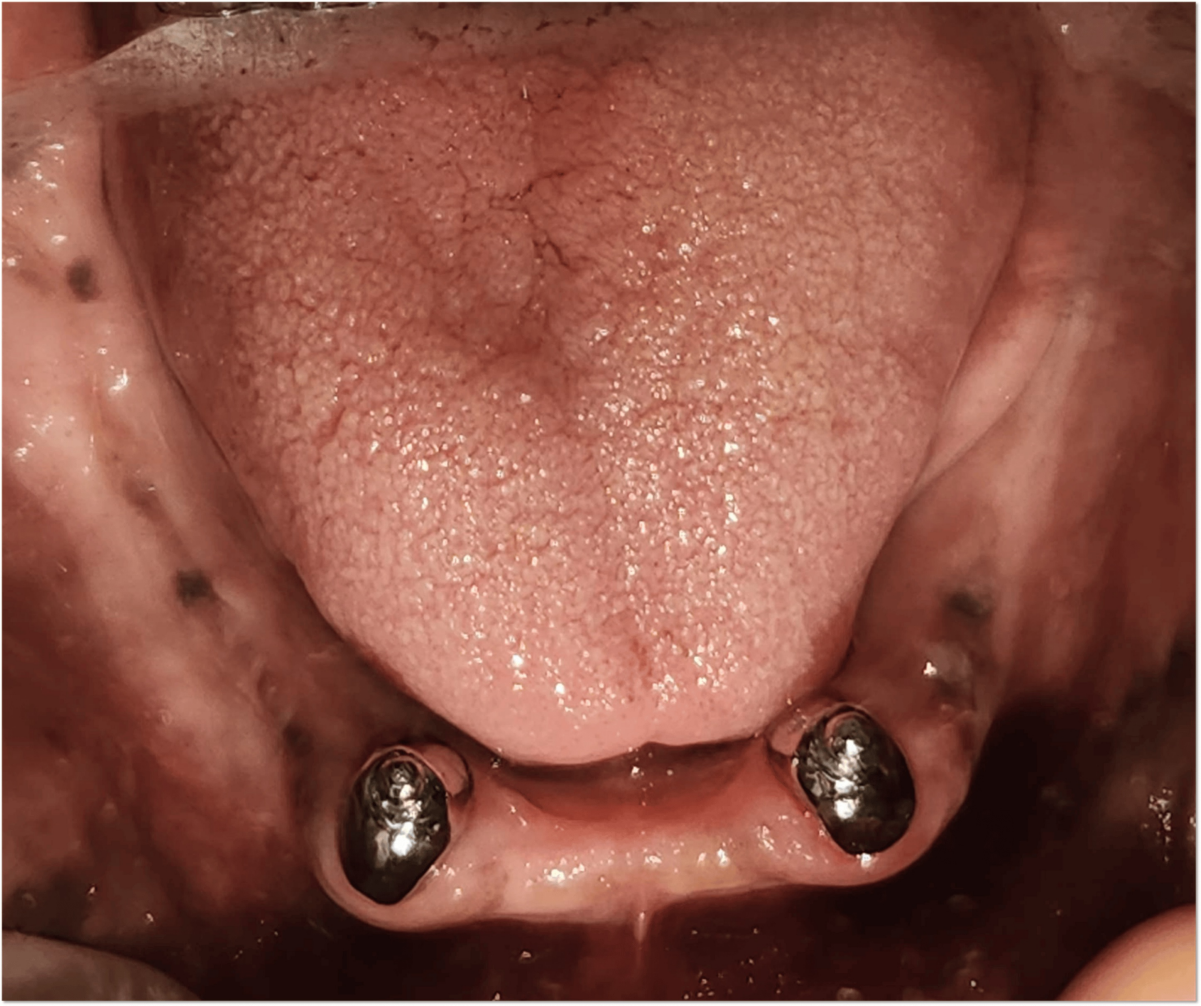
Cementation of metal coping with ball attachment i.r.t., 33 and 43

Tooth preparation was carried out for 33 and 43 by reducing the clinical crown height to create dome-shaped abutments (Figure 5). Later final impression was made with single-step putty and light body (Figure 6). Ball attachments (male component) were cemented using GIC luting cement on 33 and 43 to provide mechanical retention for the overdenture (Figure 7).

**Figure 8:**
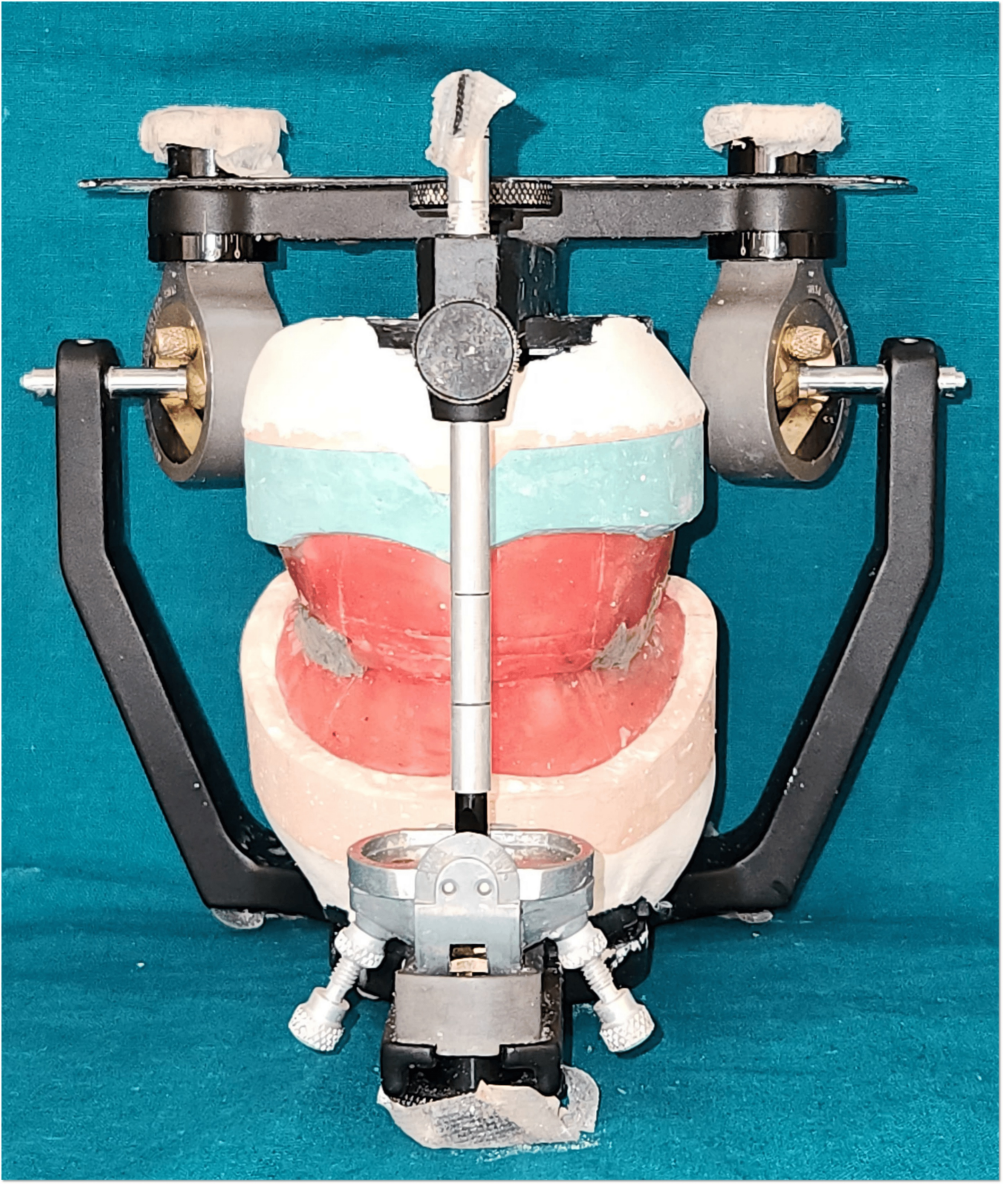
Mounted in Hanau semi-adjustable articulator

Custom trays were fabricated, and border molding was performed using greenstick compound. Secondary impressions were recorded using zinc oxide eugenol paste and light body for maxillary and mandibular arches, respectively. The master cast was poured using type III and IV for maxillary and mandibular arches, respectively. Record bases and occlusion rims were fabricated. Vertical dimension and centric relation were established using the nick and notch method. Facebow record was done and was transferred to the Hanau semi-adjustable articulator and mounted (Figure 8).

**Figure 9:**
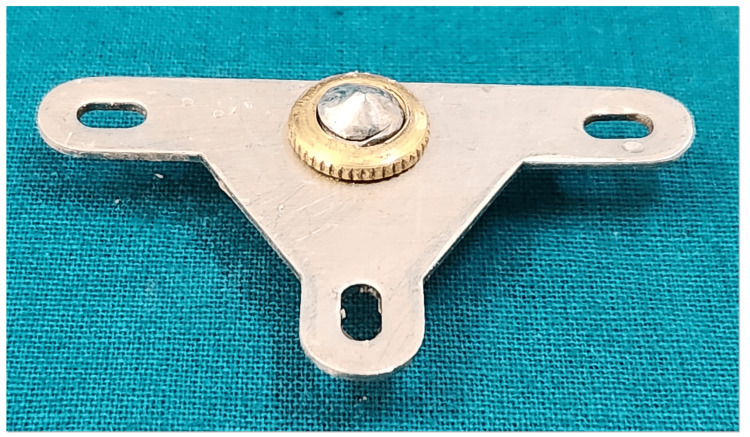
Customized intraoral tracer

A customized intraoral gothic arch tracer was fabricated for centric relation recording. The tracer assembly consisted of a stylus fitted with an adjustable screw mechanism on the mandibular record base. This customization allowed fine-tuning of the stylus height and ensured accurate tracing of mandibular movements with improved precision and stability (Figure 9).

**Figure 10:**
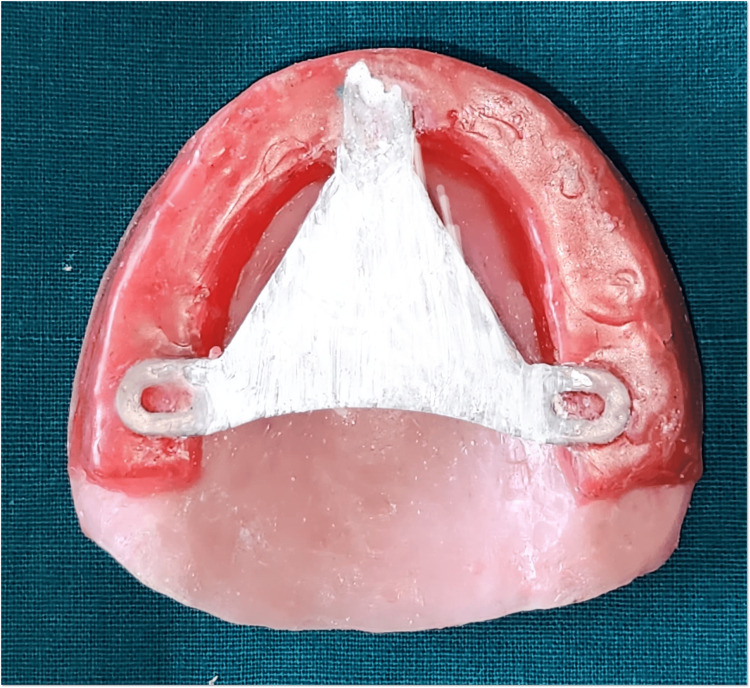
Central bearing device attached to maxillary rim

**Figure 11:**
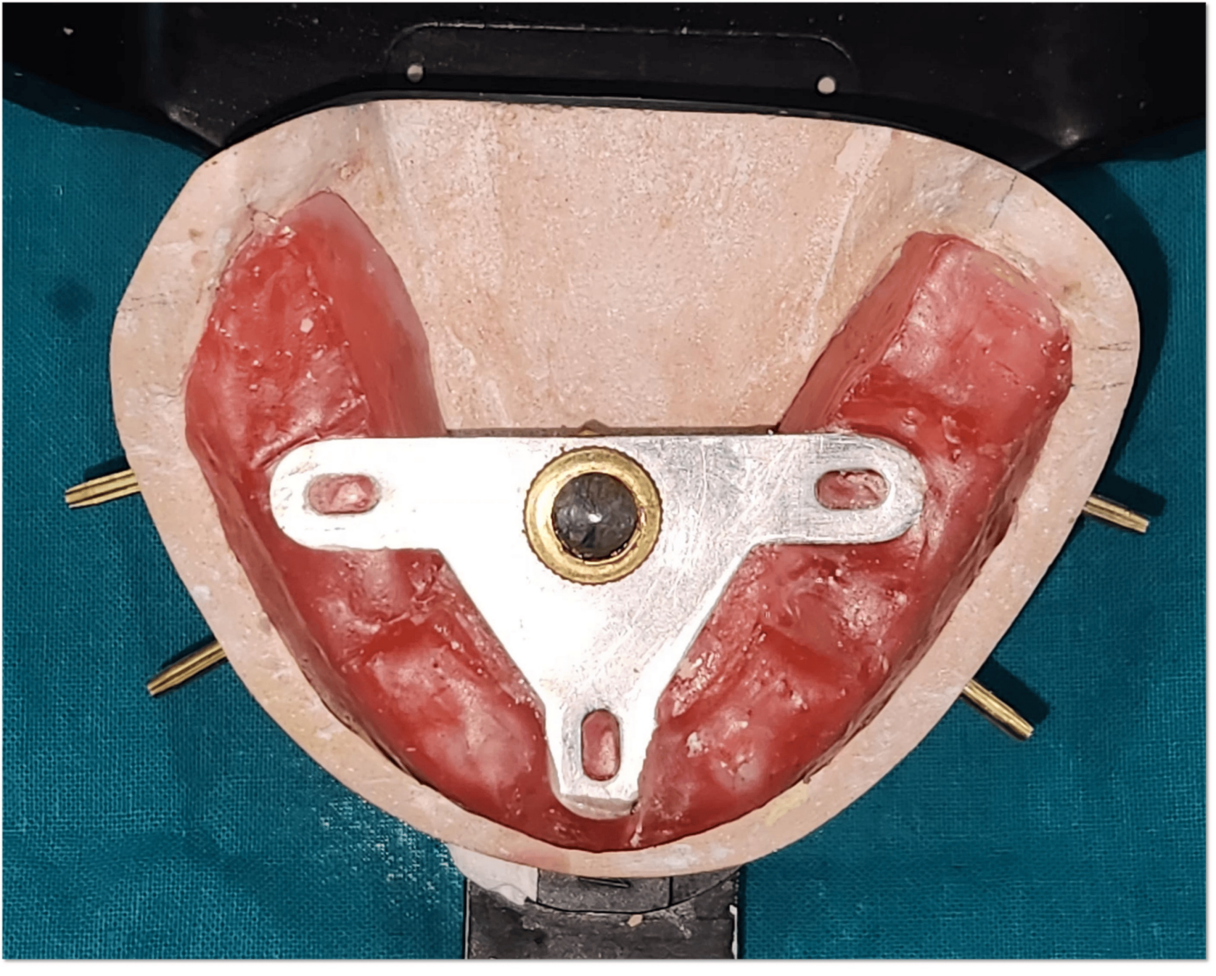
Customized stylus attached to mandibular rim

Intraoral tracer consists of a central bearing plate, a flat horizontal plate usually attached to the maxillary occlusal rim or denture base (Figure 10), and a stylus (or tracer point), which is a pointed device attached to the mandibular occlusal rim or denture base (Figure 11).

**Figure 12:**
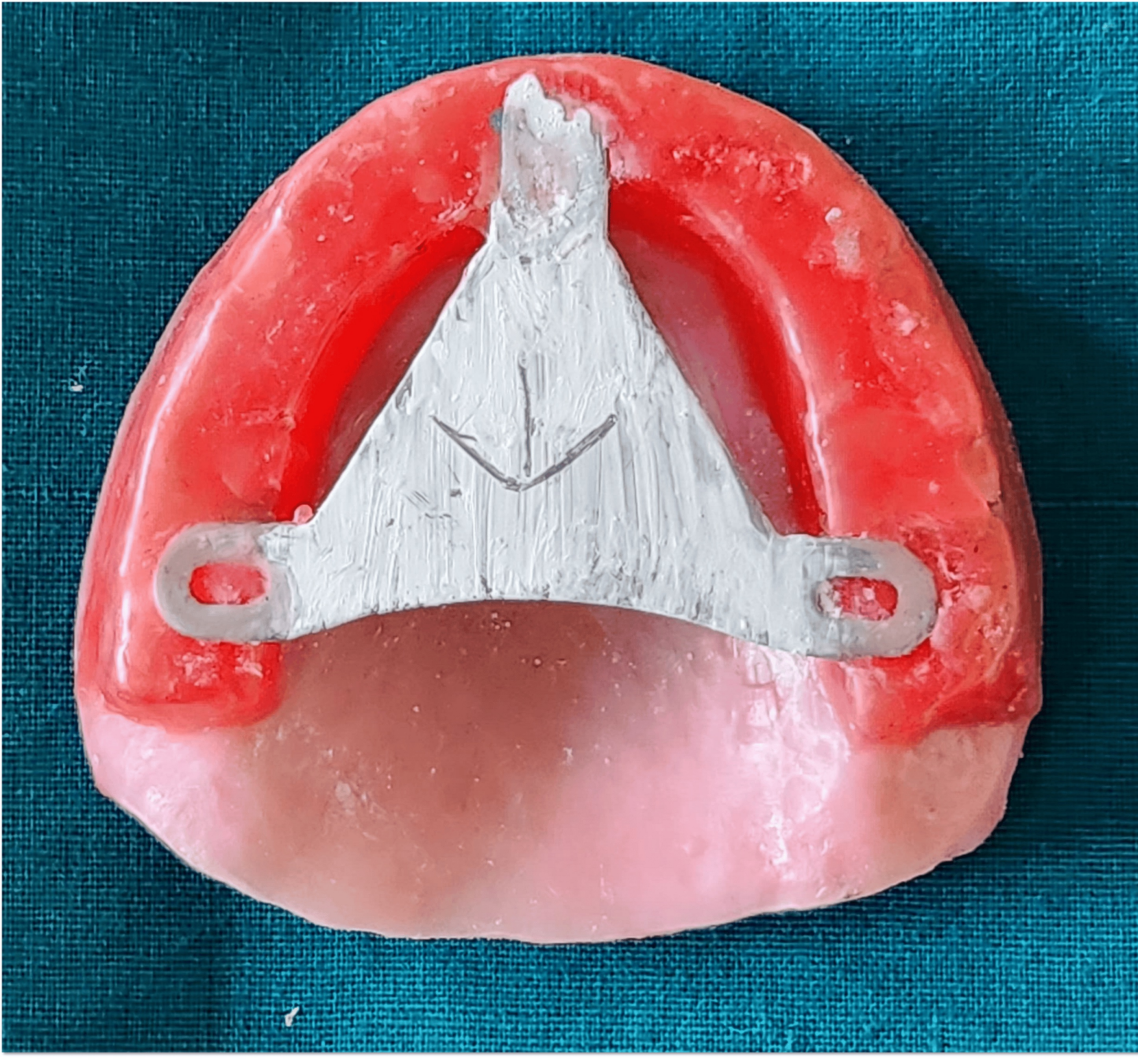
Arrow point tracing

The patient was guided to perform specific mandibular movements such as protrusive, retrusive, and right and left lateral movements. These movements together produce a characteristic Gothic arch tracing, with the apex representing the centric relation position. The typical Gothic arch tracing is shown in Figure 12.

Later, centric and protrusive interocclusal records were made. Using these records, programming of the articulator was done, horizontal guidance obtained was 25 on both sides. Applying the formula L = H/8 + 12, the calculated lateral condylar guidance (Bennett angle) was 15°.

**Figure 13:**
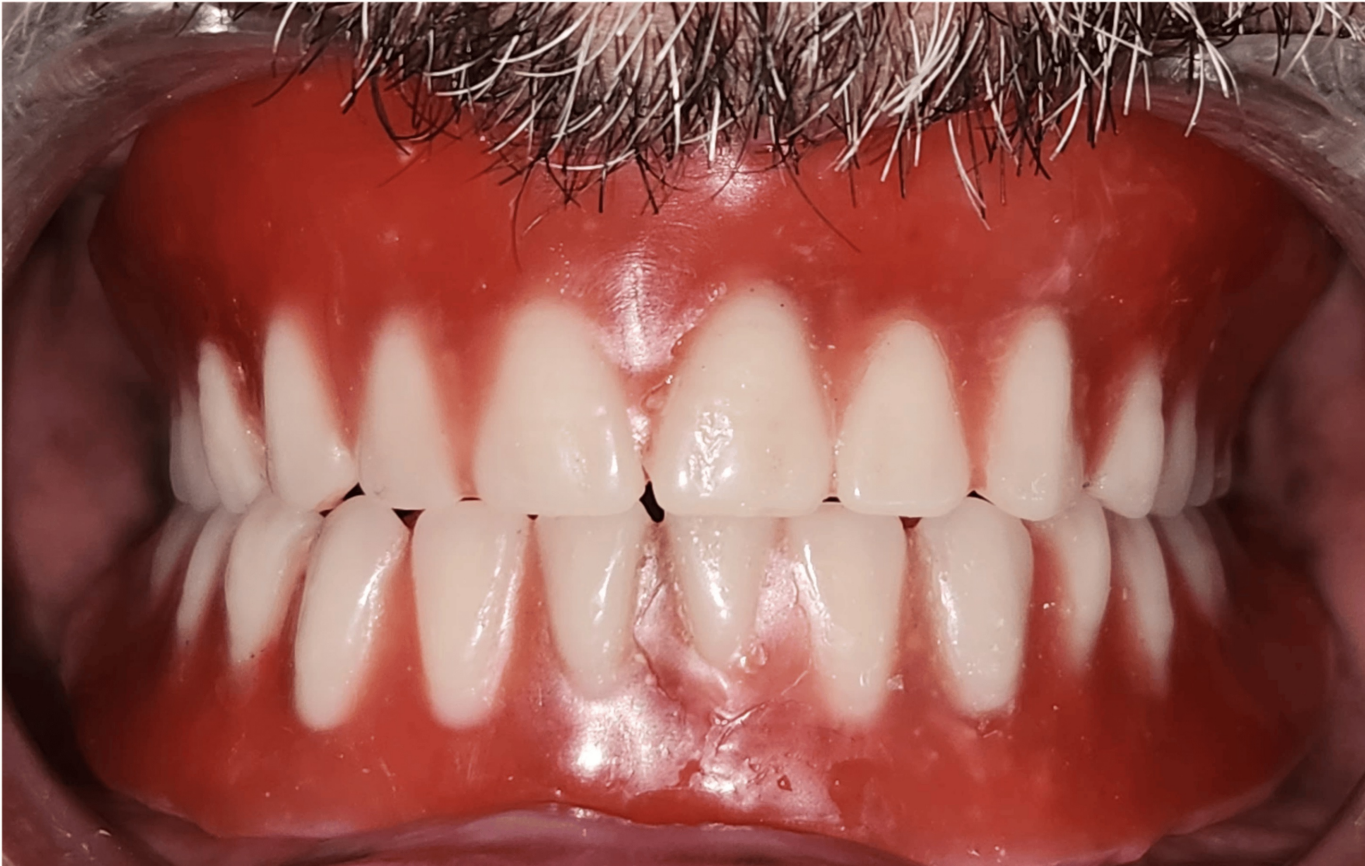
Try-in

Teeth were arranged in a bilateral balanced occlusal scheme. A try-in appointment was conducted to assess esthetics, phonetics, occlusion, and vertical dimension (Figure 13). The occlusal contacts were carefully assessed in centric relation, as well as during right and left lateral and protrusive movements.

**Figure 14:**
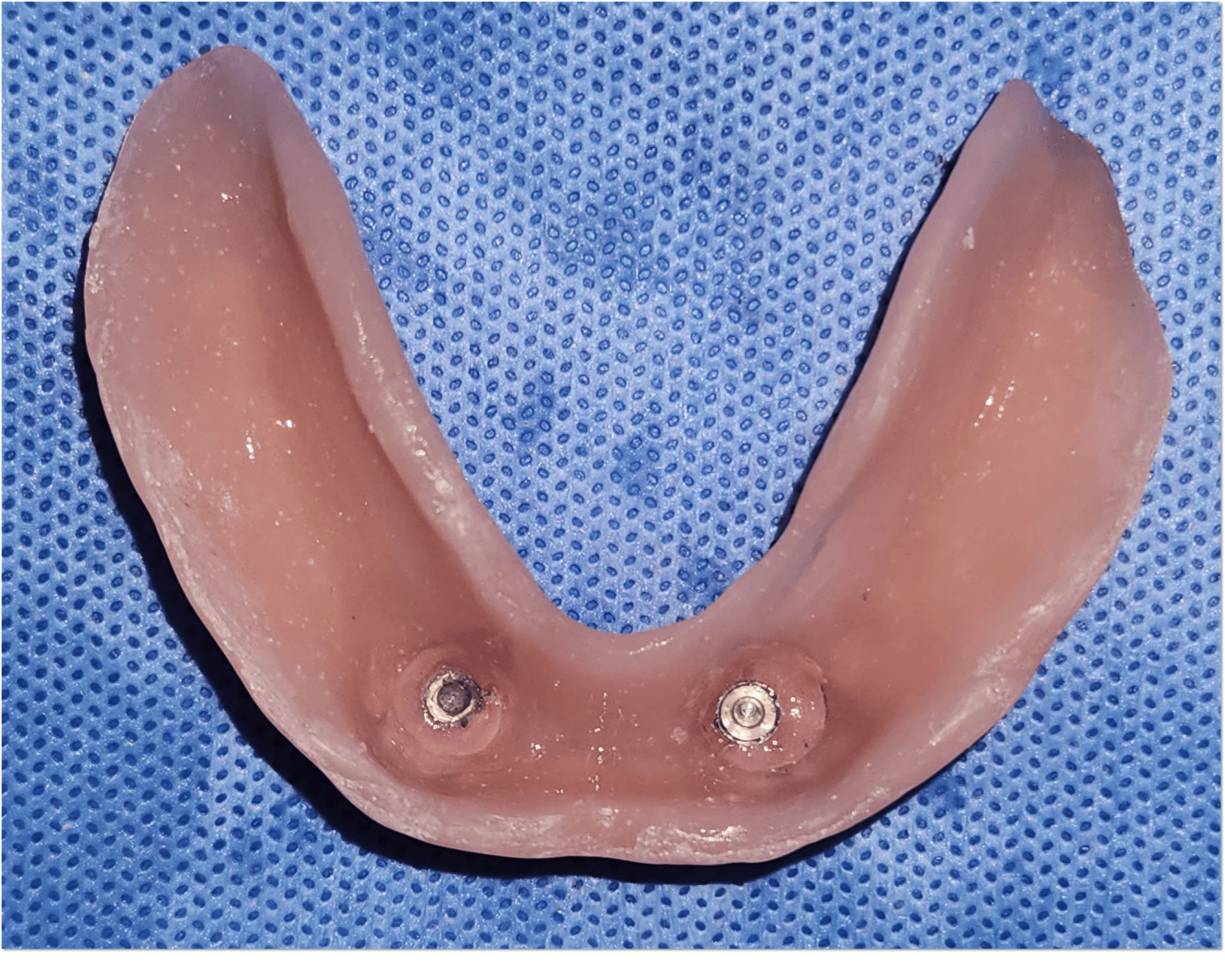
Pick-up impression

Following trial approval, the dentures were processed using heat-cured acrylic resin. During insertion, the ball attachment housings (female component) were incorporated into the intaglio surface of the mandibular overdenture (Figure 14).

**Figure 15:**
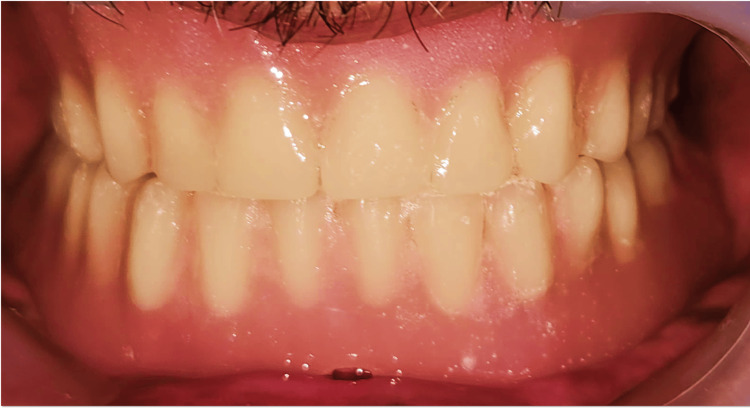
Balanced complete denture insertion

Final prosthesis insertion was done, ensuring proper seating and engagement of the attachments (Figure 15). The occlusal contacts were carefully assessed in centric relation, as well as during right and left lateral and protrusive movements. Occlusal adjustments were made to refine balanced occlusion. Post-insertion instructions were given to the patient, who reported satisfaction with the denture’s retention, stability, and esthetics. Follow-up visits at 24 hours, one week, one month, and six months revealed satisfactory clinical outcomes.

## Discussion

For the fabrication of a complete denture, adequate support relies heavily on the residual alveolar ridge. Following extraction, the alveolar bone undergoes continuous resorption, which is accelerated in the absence of functional periodontal ligaments. Retaining natural teeth with healthy periodontal support helps maintain bone volume and ridge form. In elderly patients with limited dentition, overdentures present a preventive prosthodontic option that safeguards alveolar bone while improving prosthesis function and patient comfort [6].

As a preventive prosthodontic approach, overdentures reduce the resorption of alveolar bone when compared to complete dentures. In a five-year clinical study conducted by Crum and Rooney, it was observed that patients rehabilitated with complete maxillary dentures and mandibular overdentures exhibited significantly less vertical alveolar bone loss (0.6 mm) than those wearing complete maxillary and mandibular dentures (5.2 mm) [7]. Similar findings were reported by Tallgren and Miller, highlighting the benefit of preserving anterior mandibular teeth for maintaining alveolar bone [8].

Tooth-supported overdentures are considered a conservative and preventive treatment approach over both conventional complete dentures and implant-supported overdentures. They offer several advantages, including preservation of alveolar bone, maintenance of periodontal proprioception, improved denture stability and support, enhanced masticatory efficiency, and increased patient comfort and psychological satisfaction [9]. Rissin et al. conducted a clinical comparative study evaluating the masticatory performance of patients with complete dentures, overdentures, and natural dentition. The results showed that complete denture wearers had a masticatory performance of 59%, overdenture wearers achieved 79%, and individuals with natural dentition demonstrated the highest performance at 90%. Overdenture wearers can masticate food effectively than complete denture wearers [10].

For tooth-supported overdentures, several treatment strategies can be employed in the management of abutment teeth, varying from simple modification of the tooth and its reduction, endodontic therapy followed by preparation with metal copings, or incorporation of attachments when sufficient interocclusal space is available. Retention can be enhanced when attachments are incorporated in the design by either redirecting occlusal forces away from weaker abutments towards soft tissue or towards stronger abutments [11]. Attachments in overdentures can be integrated either by affixing them to cast abutment copings or through intra-radicular placement. Careful evaluation of available vertical space is important during planning to ensure sufficient space for attachments along with an adequate denture thickness and denture teeth, without compromising the structural durability of the prosthesis [12].

Overdenture attachments are categorized as either studs, linking the prosthesis to individual teeth, or bars, connecting the prosthesis to splinted abutment teeth or magnets [13]. In this report, ball attachment systems were utilized, where the male components connected to the abutments, and the female components were housed within the intaglio surface of the denture through the use of sleeves. The ball and socket attachment allows rotation of the overdenture, thereby preserving the structural durability of the prosthesis. This system provided superior prosthetic retention and stability. In completely edentulous patients, bilateral balanced occlusion (BBO) confers better occlusion, since it promotes better masticatory efficiency and allows a greater distribution of contacts between the dentures during each masticatory movement; it also provides greater retention and stability [14].

Intraoral tracing offers precise registration of mandibular movements because the tracing occurs close to the condylar rotational axis, and the oral musculature remains relatively passive during recording. When combined with a balanced occlusion scheme, intraoral tracing can significantly enhance denture stability and support. However, this technique has certain limitations: it is not feasible in patients with severely resorbed ridges, tongue abnormalities such as ankyloglossia or macroglossia, or impaired neuromuscular control. Additionally, due to the small size of the tracing device, the recording cannot be directly visualized or continuously monitored during the procedure.

Mandibular tracing using an intraoral device offers precise and accurate registration since it is located near the axis of condylar rotation, and the oral musculature remains relatively passive during recording. Integrating balanced occlusion with intraoral tracing significantly enhances the support and stability of the prosthesis. However, intraoral tracing is not feasible in patients with severely atrophic ridges, abnormalities of the tongue like macroglossia and ankyloglossia, or impaired neuromuscular conditions. Its limitations include the small size of the intraoral device and the inability to directly visualize the recording [15]. In this case, the patient presented with well-formed ridges, which facilitated intraoral tracing and the subsequent establishment of a balanced occlusion. Balanced occlusion aims to maintain simultaneous bilateral contacts of teeth during centric and eccentric mandibular movements, thereby enhancing prosthesis stability and minimizing tipping forces [16].

## Conclusions

Tooth-supported overdentures, when planned and executed meticulously, offer significant advantages in preserving the residual ridge and enhancing the functional and psychological outcomes of prosthodontic rehabilitation. The use of ball attachments improves prosthesis retention, while the incorporation of customized intraoral tracing and balanced occlusion ensures optimal stability and functional harmony. This report demonstrates the importance of preserving remaining natural teeth and highlights the role of careful clinical planning in achieving successful long-term rehabilitation outcomes.
